# Visualization of Polymer–Surfactant Interaction by Dual-Emissive Gold Nanocluster Labeling

**DOI:** 10.3390/bios12090686

**Published:** 2022-08-26

**Authors:** Jiaojiao Zheng, Jing Zhang, Fengniu Lu, Yi Du, Ding Cao, Shui Hu, Yang Yang, Zhiqin Yuan

**Affiliations:** 1 State Key Laboratory of Chemical Resource Engineering, College of Chemistry, College of Material Science and Engineering, Beijing University of Chemical Technology, Beijing 100029, China; 2 Department of Chemistry and Chemical Engineering, Beijing Institute of Technology, Beijing 100081, China; 3 Analysis Center, Key Laboratory of Bioorganic Phosphorus Chemistry and Chemical Biology (Ministry of Education), Department of Chemistry, Tsinghua University, Beijing 100084, China; 4 State Key Laboratory of NBC Protection for Civilian, Beijing 102205, China

**Keywords:** polymer-surfactant interaction, visualization, fluorescence, gold nanoclusters, ratiometric analysis

## Abstract

Polymer-surfactant interaction decides the performance of corresponding complexes, making its rapid and intuitionistic visualization important for enhancing the performance of products and/or processing in related fields. In this study, the fluorescence visualization of the interaction between cationic hyperbranched polyethyleneimine and anionic sodium dodecyl sulfonate surfactant was realized by dual-emissive gold nanocluster labeling. The sensing mechanism was due to the interaction-induced polymer conformation change, which regulated the molecular structure and subsequent photoradiation process of the gold nanoclusters. All three inflection points of the interactions between the polymers and the surfactants were obtained by the change in fluorescence emission ratio of the designed dual-emissive gold nanoclusters. Moreover, these inflection points are verified by the hydrodynamic diameter and zeta potential measurements.

## 1. Introduction

Polymer-surfactant complexes have superior performances and are widely used in the development of detergents, imaging agents, pharmaceuticals, and functional nanomaterials [1,2,3,4]. However, the performances of these products are primarily decided by polymer-surfactant interaction [5,6]. Multiple interaction modes have been identified between polymers and surfactants [7]. Polymers and surfactants with conspecific charge show a relatively simple interaction force, and can be well described with a foam model [2]. In contrast, oppositely charged polymers and surfactants possess complicated interaction pathways, which have attracted growing attention in recent years [8]. For example, Hussain et al. investigated the interaction between poly(1,4-bis(6-(1-methylimidazolium)-hexyloxy)-benzene bromide) (PMI, water-soluble cationic conjugated polyelectrolyte) and anionic surfactants (viz., sodium dodecyl benzenesulfonate (SDBS), and sodium dodecyl sulfate (SDS)), and applied this interaction to anionic surfactant removal [9]. As indicated in previous reports, when the oppositely charged surfactant is added into a polymer solution, the strong electrostatic attraction leads to the decrease in polymer surface charge until the formation surfactant micelle aggregates; such a surfactant concentration is called as critical aggregation concentration (CAC) [8,10]. The increase in surfactant neutralizes polymer charge and causes the formation of precipitation, which is denoted as charge neutralization concentration (CNC) [8]. Further addition of the surfactant results in the reversion of surface due to its hydrophobicity and dispersion of the complex, which adsorbs the surfactant until saturation, known as the polymer saturation point (PSP) [11]. Therefore, the visualization of these critical points is significant to understanding the polymer-surfactant interaction and to enhance the performance of products and/or processing in related fields.

Toward this goal, many techniques have been developed for revealing the interaction of oppositely charged polymers and surfactants in solution phase, such as surface tension, viscosity, electroconductivity, zeta potential, dynamic light scattering, and fluorimetry [12,13,14,15,16,17]. For example, Yang et al. achieved the imaging of the CAC and PSP points of the sodium carboxymethyl cellulose-stepanol WA-100 system [18]. Among these techniques, the fluorimetry-based ones have attracted wide interest because of their high sensitivity and low background [19,20]. For instance, Jiao et al. reported a fluorescence imaging tool that illustrates the CAC and PSP points of chitosan and tetraphenylethene-modified sodium dodecyl sulfonate surfactant (TPE-SDS) [21]. However, it is still difficult to illustrate all the critical points intuitively. During polymer–surfactant interaction, the dramatic structural change in the polymer cannot be ignored [22]. Thereby, it is possible to enhance the resolution and visualize all critical points by fluorescence labeling in the polymer backbone.

**Figure 1:**
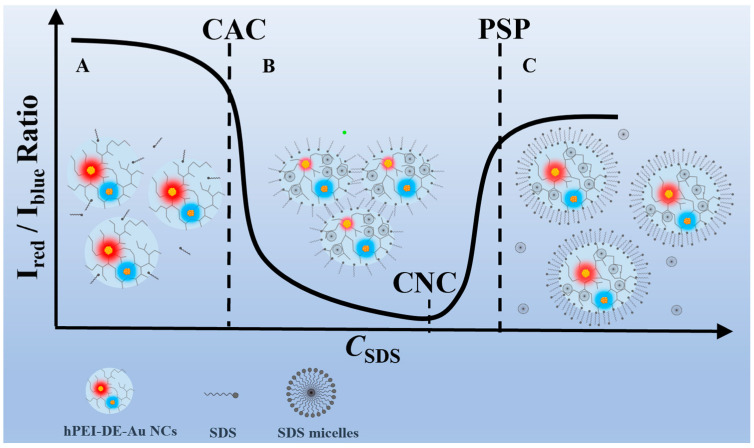
Schematic illustration of hPEI-SDS interaction visualization by DE-AuNC labeling. hPEI-DE-Au NCs interacting with 0–1.6 mM SDS (A), 1.6–8 mM (B), and 8–10 mM (C).

Gold nanoclusters (Au NCs) with bright emission have been widely investigated in sensing, imaging, and antibacterial agents, etc. [23,24,25,26,27,28,29,30]. In recent years, we found that the cationic polymer (hyperbranched polyethyleneimine, hPEI) can act as an effective template to produce fluorescent Au NCs and silver NCs [31,32]. With control of the hPEI amount, Au NCs with tunable blue and red dual emission (DE-Au NCs) could be produced [33,34,35]. Taking this factor into consideration, it is thus speculated that DE-Au NC labeling could be used for visualizing the hPEI-surfactant interaction, which is theoretically possible. As a proof of concept, we herein explored a fluorimetric strategy to investigate the hPEI-SDS (a typical anionic surfactant) interaction through DE-Au NC labeling. Interestingly, the DE-Au NCs showed a temperature-responsive emission ratio, which was attributed to the hPEI conformation change-mediated variation in molecular structure and the subsequent photoradiation process. With DE-Au NC labeling, all three critical points of the hPEI-SDS system were observed, as illustrated in Figure 1. The accuracy was also verified with the hydrodynamic diameter and zeta potential measurements. In brief, we proposed a simple fluorimetric approach to realize the visualization of all three critical points of the polymer–surfactant system by DE-Au NC labeling.

## 2. Materials and Methods

### 2.1. Chemicals and Materials

Chloroauric acid tetrahydrate (HAuCl_4_·4H_2_O) was purchased from Adamas-beta (Shanghai, China). Hyperbranched polyethyleneimine (hPEI, Mw 25000) was purchased from Shanghai Macklin Biochemical Co., Ltd. (Shanghai, China). 11-Mercaptoundecanoic acid (MUA) was purchased from Shanghai Yuanye Bio-Technology Co., Ltd. (Shanghai, China). L-ascorbic acid (AA) was obtained from Hunan Intellijoy Biotechnology Co., Ltd. (Changsha, China). Fluorescein sodium salt (FlS) was purchased from Shanghai Macklin Co., Ltd. (Shanghai, China). Sodium dodecyl sulfonate (SDS) was purchased from TCL Co., Ltd. (Shanghai, China). Ethanol, hydrochloric acid (HCl), and sodium hydroxide (NaOH) were purchased from Beijing Chemical Reagent Company (Beijing, China). All the chemicals used were analytical-reagent grade and used without further purification. Ultrapure water (18.2 MΩ) was obtained from a Millipore system. All glassware was cleaned by fresh aqua regia.

### 2.2. Apparatus

The UV-vis absorption spectra were collected using a UV−3900H spectrophotometer (Shimadzu, Tokyo, Japan). Fluorescence spectra were obtained using an F-7000 fluorescence spectrophotometer (Hitachi, Tokyo, Japan) at a slit of 5.0 nm with a scanning rate of 2400 nm/min. Zeta potential and hydrodynamic diameter were determined using a Malvern Zetasizer 3000 HS nano-granularity analyzer (Malvern, UK). Transmission electron microscopy (TEM) was performed on an HT7700 transmission electron microscope (HITACHI, Japan). The pH values were measured using a benchtop pH meter (Orion plus, Thermo Fisher, Waltham, MA, USA). The time-resolved fluorescence decay curve was obtained on FLS 980 (Edinburgh, UK) and measured by exciting at 310 nm with a microsecond flashlamp. The fluorescence intensity of the measured sample had a certain attenuation law after being excited by the light pulse, after which the fluorescence intensity attenuation curve of each point in the measured sample was fitted and analyzed. The fluorescence lifetime value (*τ*) was obtained by the following lifetime formula:

$$\tau =\frac{\tau_{1}^{2}B_{1}+\tau_{2}^{2}B_{2}+\tau_{3}^{2}B_{3}}{\tau_{1}B_{1}+\tau_{2}B_{2}+\tau_{3}B_{3}}$$

where *τ*_1_, *τ*_2_, and *τ*_3_ are the lifetimes of fitted components and *B*_1_, *B*_2_, and *B*_3_ are the corresponding integrate area of fitted components.

### 2.3. Synthesis of DE-Au NCs

The DE-Au NCs were synthesized according to our previous report with slight modifications [33]. Typically, 50 μL of HAuCl_4_ (0.1 M) was dissolved in 3.75 mL of ultrapure water at 25 °C, then was mixed with 50 μL of hPEI (10 mM) to ensure the hPEI/HAuCl_4_ molar ratio of 1:5. After 10 min stirring, the solution turned into a brown-yellow color, indicating the entire complexation between Au (III) and hPEI. The addition of 50 μL of AA (0.1 M) induced the gradual color fading within 15 min. At this point, 100 μL of MUA ethanol solution (0.1 M) was introduced into the colorless solution. The solution was stirred at room temperature for another 6 h; the obtained clear bright yellow solution emitted a dual emission under UV light irradiation. Finally, the DE-Au NC solution was filtered by a filter membrane of 0.22 μm to remove any unwanted reactants or byproducts. A series of DE-Au NCs with different red-blue fluorescence ratios were synthesized by changing the addition amount of hPEI to adjust the molar ratio of hPEI to HAuCl_4_ (from 1:20 to 5:1). The as-synthesized DE-Au NCs were stored at room temperature before further characterization and application.

### 2.4. Temperature-Induced Fluorescence Response Measurement

For investigating the temperature-induced fluorescence variation, the emission spectra of DE-Au NCs were recorded under various temperatures. The temperature of the DE-Au NC solution was controlled by a thermal-mixer, after 10 min stabilization, the fluorescence emission spectra of the DE-Au NCs were measured usign an F-7000 fluorescence spectrophotometer with 310 nm excitation. For reversible temperature effect study, the DE-Au NC solution was first heated to 70 °C, then the solution natural cooled to 20 °C, and the cycle was repeated with same protocol. With 10 min equilibrium, the fluorescence emission spectra were recorded using a F-7000 fluorescence spectrophotometer under 310 nm excitation.

### 2.5. hPEI-SDS Interaction Measurement

To visualize the hPEI-SDS interaction, the fluorescence emission spectra of the DE-Au NCs were measured in the presence of SDS. Typically, SDS was added to the diluted DE-Au NC (final hPEI concentration was 66.7 μM) solution, the final concentrations of SDS ranged from 0 to 10 mM. With 10 min equilibrium, the fluorescence emission spectra were recorded using a F-7000 fluorescence spectrophotometer under 310 nm excitation. The conventional measurements of the hPEI-SDS system were conducted as follows: hPEI solution (66.7 μM) was mixed with various SDS concentrations (from 0 to 10 mM), after 10 min equilibrium, the hydrodynamic diameter and zeta potential of the mixed solutions were measured with a Malvern Zetasizer 3000HS nano-granularity analyzer.

## 3. Results and Discussions

### 3.1. Synthesis and Characterization of DE-Au NCs

**Figure 2:**
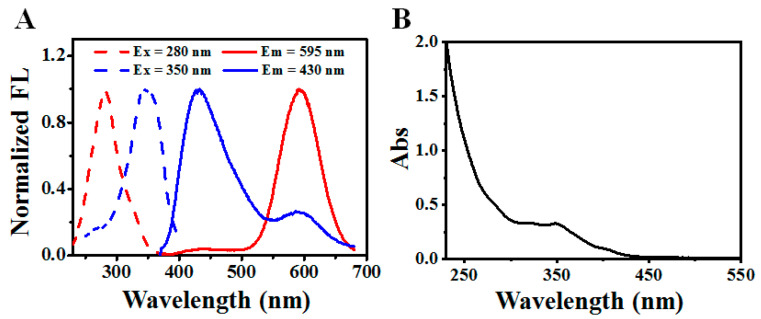
Fluorescence excitation and emission spectra (**A**) and UV-vis absorption spectra (**B**) of as-prepared DE-Au NCs.

The DE-Au NCs were synthesized by a one-pot and two-step approach from our previous report with slight modification and were characterized with UV-vis absorption spectrometry, steady-state and time-resolved fluorescence spectrometry. With a 2 h reaction, the solution changed from a brown-yellow color to colorless and finally light yellow, indicating the conversion of reactants to products. The resulted solution, however, emitted blue and red emission under 365 nm and 254 nm excitation, respectively. The dual-emission color character suggests the successful preparation of the DE-Au NCs. As shown in Figure 2A, two fluorescent components with maximum excitation/emission pairs around 350/430 nm and 280/595 nm were observed, which are consistent with previous results and indicates the generation of DE-Au NCs. According to the UV-vis absorption spectra (Figure 2B), characteristic absorption peaks located at 350 nm and 280 nm appeared, which belong to the blue-emissive and red-emissive components, respectively. The absence of absorption peak around 520 nm ruled out the generation of large plasmonic gold nanoparticles [36,37]. As referred in previous work, the absorbance of 350 nm is assigned for the d-sp electron transition of blue-emissive Au_8_ NCs (B-Au NCs), while the absorbance of 280 nm is ascribed to the ligand to metal–metal charge transfer (S→Au···Au) of red-emissive Au NCs (R-Au NCs) [33,34]. The obvious dual-emission colors, as well as the characteristic absorption peaks, confirm the successful production of DE-Au NCs.

### 3.2. Temperature-Induced Ratiometric Change in DE-Au NCs

The bright fluorescence and dual-emission characters suggest the great potential for developing ratiometric probes with the prepared DE-Au NCs [38,39,40,41]. In general, the key point of the polymer-surfactant interaction monitoring is to reveal the conformation change in the polymer [42]. The molecular thermal motion changes the polymer structure naturally and leads to certain conformation changes [43]. In this case, the investigation of the temperature-responsive character is significant for illustrating the polymer-surfactant interaction process.

**Figure 3:**
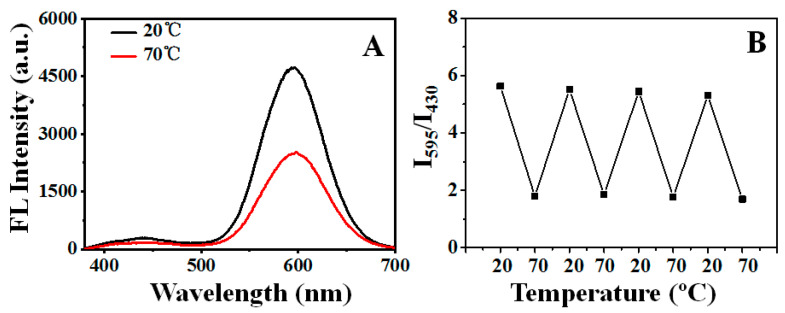
(**A**) Fluorescence emission spectra of DE-Au NCs under various temperatures. (**B**) Reversible temperature dependence of the fluorescence intensity ratio (I_595_/I_430_) of DE-Au NCs.

It was seen that the red emission dramatically decreased upon increasing the temperature from 20 to 70 °C (Figure 3A). In contrast, the blue emission only showed a slight decrement with the same temperature difference. As a result, a ratiometric variation in the DE-Au NCs could be obtained by adjusting the temperature. Interestingly, the change in the fluorescence intensity ratio was reversible (Figure 3B), indicating that the temperature-mediated fluorescence variation is not ascribed to the destruction of Au NCs, but the change in the surrounding environment.

**Figure 4:**
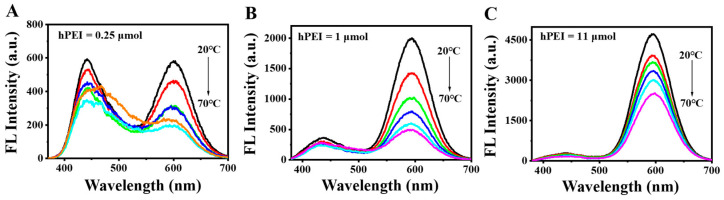
Temperature-dependent fluorescence emission spectra of the DE-Au NCs synthesized with the addition of 0.25 μmol (**A**), 1 μmol (**B**), and 11 μmol (**C**) hPEI, respectively.

It was noticed that the addition of hPEI largely affects the compositions of both the red Au NCs and blue Au NCs, and it is thus assumed that the hPEI content may influence the responsive sensitivity. To investigate the hPEI content effect, the DE-Au NCs were synthesized by changing hPEI contents from 0.25 to 25 μmol. As shown in Figure S1A, the red emission increased with the increasing hPEI content from 0.25 to 11 μmol and then decreased with a further increase in the hPEI content to 25 μmol (Figure S1B). On the other hand, the blue emission gradually decreased with the increasing hPEI content. The fluorescence ratio showed a rapid increment at the hPEI content of 1 μmol and a maximum at the 11 μmol hPEI content. On the basis of the fluorescence ratio trend (Figure S1C), to avoid the false results, a high hPEI content (>11 μmol) could not be chosen, partially due to the ultra-weak blue emission. To find out the optimal hPEI content, temperature-responsive fluorescence emission of three of the DE-Au NCs by 0.25, 1, and 11 μmol hPEI templates were tested. As manifested in Figure 4, the red emission gradually decreased in all three of the DE-Au NCs when the temperature changed from 20 to 70 °C with an interval of 10 °C, indicating that the hPEI content did not change the temperature-responsive nature of the DE-Au NCs. However, a low hPEI content led to a weak red emission, while a high hPEI content resulted in an unobvious blue emission. Such unmatched emission components may not be able to yield sensitive ratiometric change. In order to study the emission variation trends versus the temperature, both the fluorescence intensity of the red emission and fluorescence intensity ratio were conducted with three of the DE-Au NCs. According to Figure S2A–C, the absolute decrement of the red emission appeared in all three of the DE-Au NCs and the maxima appeared with the 11 μmol hPEI content. Meanwhile, the fluorescence intensity ratio (I_595_/I_430_) of all DE-Au NCs decreased with the increasing temperature (Figure S2D–F) and the minimum variation appeared with the 11 μmol hPEI content. A possible reason is that a high hPEI content leads to a weak blue emission in spite of the strong red emission and thus generates a high intensity ratio value and a small variation. When the hPEI content was 1 μmol, the fluorescence intensity ratio variation showed the highest slope and the I_595_/I_430_ value was larger than 2. In this case, the DE-Au NCs with 1 μmol hPEI, showing a high response sensitivity, were chosen for the subsequent experiments.

### 3.3. Mechanism of Temperature-Responsive Fluorescence of DE-Au NCs

The different variation trends of the two components suggest that the fluorescence quenching is not caused by molecular thermal motion only, otherwise, the quenching behaviors should be consistent at the same temperature. As reported in our previous works, the fluorescence of the R-Au NCs is originated from the LMMCT, while the fluorescence of the B-Au NCs is produced from the d-sp electron transition [33]. The former is distinctly affected by the Au(I)–MUA motif, including the MUA density, the electron donating capability of sulfur atoms, and the MUA rotation [44,45]. While the latter is primarily related to the Au atom number of the Au NCs, the surface MUA shows an ignorable contribution. Therefore, we hypothesized that the temperature-induced distinct fluorescence variations in the R-Au NCs and B-Au NCs might be assigned to the hPEI conformation change, which leads to the spatial displacement of the surface MUA. The varied spatial position of the surface MUA alters the S→Au···Au charge transfer pathway of the R-Au NCs, while it has no influence on the B-Au NCs. As a consequence, only the red emission of the R-Au NCs decreases significantly with the increase in temperature.

**Figure 5:**
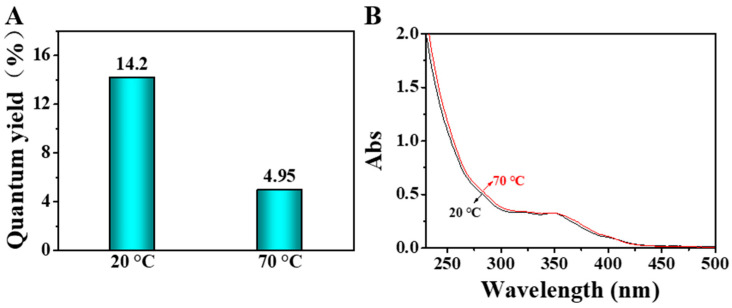
Quantum yields (**A**) and UV-visible absorption spectra (**B**) of DE-Au NCs at 20 and 70 °C, respectively.

To verify this conjecture, we first measured the fluorescence quantum yield (QY) and the UV-vis absorption spectra of the DE-Au NCs at different temperatures. As shown in Figure 5A, the QY of DE-Au NCs at 20 °C was determined to be 14.2%, while it became 4.9% at 70 °C. The decreased QY at a high temperature indicates the decrease in the irradiative transition contribution. It was seen that the absorbance around 270–280 nm slightly increased upon heating the DE-Au NC solution from 20–70 °C (Figure 5B). Such an increment suggests that the LMMCT process can be affected by temperature. To further confirm the temperature-responsive LMMCT process, the time-resolved fluorescence emission spectra of the R-Au NCs at different temperatures were obtained. When the temperature rose from 20 to 40 and then to 70 °C, the fluorescence lifetime of the R-Au NCs changed from 4.206 to 2.712, and then to 1.334 μs (Figure S3). Interestingly, the fluorescence lifetime changed to 4.479 μs when the temperature was down to 20 °C. As is known, excited molecules deactivate to a ground state or low-energy excited state by releasing energy through an emissive pathway and vibration process/thermal exhaustion, also called radiative transition and non-radiative transition [46,47]. These two transitions can be described using the following equations: *k*_r_ = *φ*/*τ*, *k*_nr_ = (1 − *φ*)/*τ*, where *k*_r_ and *k*_nr_ are the rate constants of radiation transition and non-radiation transition, *φ* is the QY, and *τ* is the fluorescence lifetime. The *k*_nr_/*k*_r_ ratio was calculated to be 6.04 at 20 °C, while it became 19.20 at 70 °C, suggesting the dramatically increased non-radiation transition at 70 °C. The decreased fluorescence lifetime, as well as the increased *k*_nr_/*k*_r_ ratio, indicates the change in the LMMCT pathway of the R-Au NCs at a high temperature.

**Figure 6:**
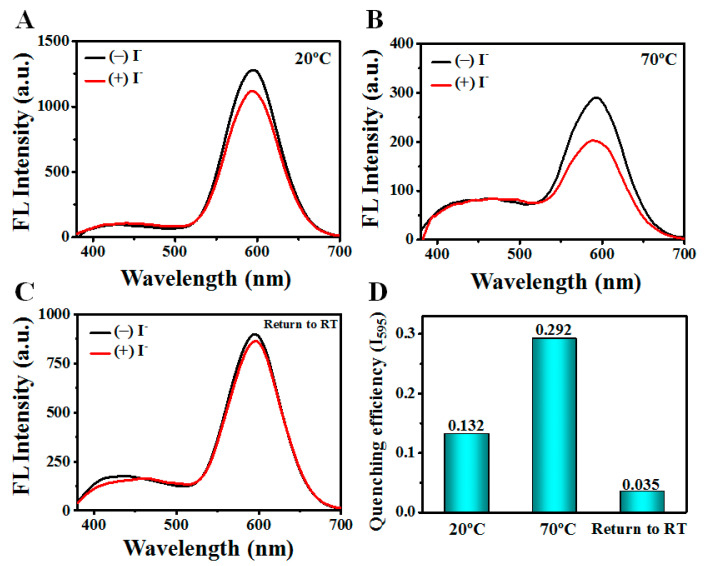
I^−^ quenching behavior investigation. Fluorescence emission spectra of DE-Au NCs in the absence (black line) and presence (red line) of I^−^ under 20 °C (**A**), 70 °C (**B**), and return to room temperature (**C**), respectively. (**D**) Relative fluorescence decreases in red emission under various conditions with the addition of I^−^.

Since the MUA density and electron donating capability of sulfur atoms have no change under the heating process, a possible cause of obvious fluorescence quenching is the MUA rotation, which changes the LMMCT efficiency and diminishes the fluorescence. Such an MUA rotation might be attributed to the temperature-induced hPEI conformation variation. It should be noticed that the hPEI conformation variation may expose the inner DE-Au NCs and change their accessibility. To compare the accessibility of DE-Au NCs, I^-^ quenching experiments were conducted at different conditions. As shown in Figure 6, the fluorescence quenching of red emission was largely enhanced at 70 °C, indicating the increased accessibility of the R-Au NCs at a high temperature. On the other hand, the blue emission was not quenched, partially due to the solid structure as referred to in previous work. The reversible accessibility suggests that the R-Au NCs are squeezed out of the hPEI networks with an increased temperature. We also tested the hydrodynamic diameter distribution of the DE-Au NCs under the same conditions. It was found that a slight decrease in the hydrodynamic diameter appeared at a high temperature (Figure S4). The alkyl chain of MUA rotated quickly with a high temperature, thus the hPEI network had to shrink to maintain a good solubility, leading to a slight decrease in the hydrodynamic diameter. For organic fluorophore, the hPEI conformation change did not show a remarkable influence on its temperature-responsive fluorescence (Figure S5), further validating the importance of the molecular variation in the fluorophores. Taken together, these results show that the temperature-induced emission variation in the DE-Au NCs is attributed to the hPEI conformation change-mediated variation in the molecular structure and subsequent LMMCT process.

### 3.4. Visualization of hPEI-SDS Interaction

The temperature alters the hPEI conformation and changes the emission ratio, making it possible to visualize the hPEI-surfactant interaction by DE-Au NC labeling. Herein, typical anionic surfactant sodium dodecyl sulfonate (SDS) was chosen to illustrate hPEI-anionic surfactant interaction. It is accepted that a strong hPEI-SDS interaction may offer distinct conformation change, thus resulting in dramatic emission ratio variation. In addition, it should be noticed that cationic hPEI and anionic SDS are oppositely charged under an appropriate pH. Before the investigation of the interaction process, the hPEI-SDS interaction was conducted under various pH solutions. It was seen that the hydrodynamic diameter of the hPEI-SDS complex was large at a pH 11 condition (Figure S6), indicating that the hPEI-SDS interaction is strong under alkaline conditions. Since hPEI possesses three amine groups (primary, secondary, and tertiary amines), it is generally positive in aqueous media. However, SDS only exhibits a high negative charge under an alkaline environment. The easily observed hydrodynamic diameter reveals a good reaction condition in an alkaline environment. In this case, all subsequent experiments were conducted at pH 11.

**Figure 7:**
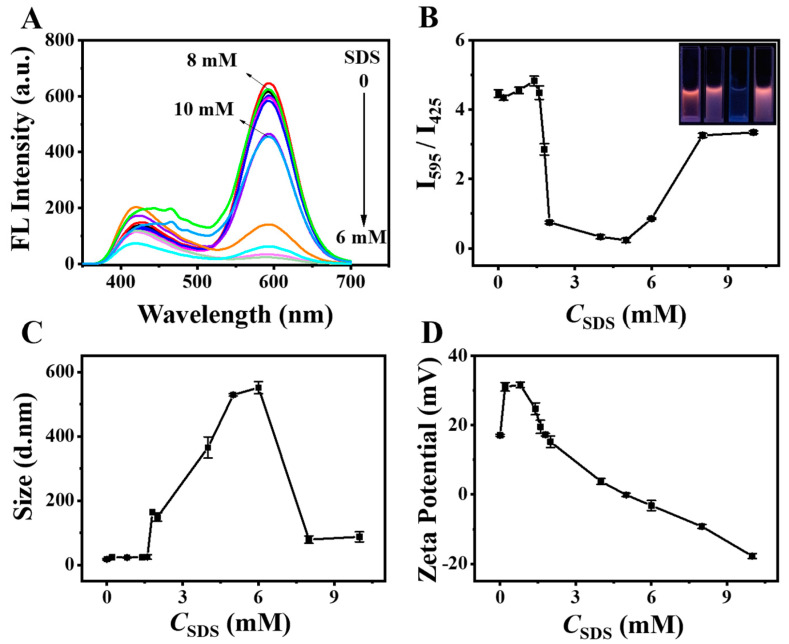
(**A**) Fluorescence emission spectra of DE-Au NC solution upon adding SDS with various concentrations. (**B**) Plots of (I_595_/I_430_) versus the SDS concentration. Inset images were the fluorescence images of DE-Au NC solution after adding SDS (from left to right: 0, 1.6, 5, 8 mM SDS, respectively) under 310 nm UV light irradiation. Hydrodynamic diameter (**C**) and zeta potential (**D**) of DE-Au NCs upon adding SDS with various concentrations.

To visualize the hPEI-SDS interaction, the fluorescence emission spectra of the DE-Au NC solution in the presence of various SDS were recorded. As shown in Figure 7A, the red emission of the R-Au NCs showed a slight decrease when the SDS concentration was lower than 1.6 mM, and it suddenly decreased with 1.6 mM SDS (CAC point) and reached the minimum at 5 mM SDS. A further increase in SDS concentration caused a dramatic increment of the red emission. In contrast, the blue emission of B-Au NCs showed small variations. The decreased red emission is largely due to the hPEI-SDS interaction-induced hPEI conformation change, which changes the LMMCT process and inhibits fluorescence. A high SDS concentration yields the formation of isolated micelle and weakens the hPEI conformation change, which results in a small fluorescence variation. Accordingly, the intensity ratio (I_595_/I_430_) was first maintained with low SDS concentrations, then diminished after 1.6 mM SDS and reached a minimum at 5 mM SDS (Figure 7B). Interestingly, it recovered after 8 mM SDS was added and reached a flat form with a high SDS concentration. The corresponding fluorescence images of the DE-Au NC solution under each stage showed dramatic variation with various SDS concentrations (Figure 7B inset), suggesting the successful visualization of the hPEI-SDS interaction by DE-Au NC labeling. As mentioned above, the oppositely charged surfactant binds to the polymer through electrostatic attraction and undergoes three critical concentration points, CAC, CNC, and PSP. The fluorescence lifetimes of different critical points were added as suggested. It was found that the fluorescence lifetime of the R-Au NCs changed from 3.268 to 3.036, 1.285, and then 3.757 μs after adding SDS with various concentrations (Figure S7). The lifetime variation trend was consistent with that of the fluorescence ratio, which supports the assumption, as referred to above. On the basis of the intensity ratio (I_595_/I_430_) curve, the CAC, CNC, and PSP points toward the hPEI-SDS system were determined to be 1.6, 5, and 8 mM, respectively. In many reported works, only CAC and PSP points are observed. The CNC point is difficult to find, partially because of the aggregation-insensitive reporters. Thus, DE-Au NC labeling is an effective strategy for visualizing the hPEI-SDS interaction process.

To further demonstrate the visualization of the hPEI-SDS interaction by DE-Au NC labeling, the hydrodynamic diameter and zeta potential of DE-Au NCs were measured with various SDS concentrations under the same conditions. As shown in Figure 7C, the hydrodynamic diameter of the DE-Au NCs showed a negligible increase when the SDS concentration was lower than 1.6 mM. At a low SDS concentration, SDS molecules sparsely adsorbed on the hPEI chain, which did not induce an obvious conformation change. Meanwhile, the surface charge became more positive as SDS was added (Figure 7D). This is significant because the encapsulation of SDS would induce the retroflexion of the hPEI molecule, which exposes wrapped amino groups. It was easily observed that the hydrodynamic diameter of the DE-Au NCs grew quickly when the SDS concentration was higher than 1.6 mM and reached a maximum at 6 mM. A further increase in SDS led to sharp decrease in the hydrodynamic diameter. At the same time, the zeta potential value showed a rapid decrease from +30 to +17 mV, indicating that the positive charge sites of hPEI have been fully exposed and gradually neutralized by the negatively charged SDS. A neutral point was obtained with a 5 mM SDS, and the further addition of SDS led to a negatively charged product. It was found that the CNC point showed a slight difference in both the fluorimetric analysis and the hydrodynamic diameter measurement. The continuous addition of SDS neutralized the surface charge and presented the CNC point. It was noticed that the nanomaterials without a surface charge easily form aggregates. As a result, the hydrodynamic diameter measurements showed a slight variation and large error bars with a 5–6 mM SDS. The slight difference between fluorimetric analysis and hydrodynamic diameter measurement was thus reasonable due to the error. Through the combination of hydrodynamic diameter and zeta potential measurements, the CAC, CNC, and PSP points were confirmed to be 1.6, 5 and, 8 mM, respectively. The TEM images showed the shape and size of the DE-Au NC solution with a 1.6 mM SDS, a 5 mM SDS, and an 8 mM SDS (Figure S8). These results validate the accuracy of the visualization of the hPEI-SDS interaction by DE-Au NC labeling. In comparison with traditional techniques, the proposed fluorimetric approach has an easy operation and a rapid response, thus providing a promising tool for investigating the polymer-small molecule interaction processes.

## 4. Conclusions

In summary, we synthesize highly fluorescent and temperature-responsive DE-Au NCs using the one-pot and two-step protocol for the fluorimetric visualization of the hPEI-SDS interaction. The temperature-induced variation in red emission is assigned to the hPEI conformation-mediated LMMCT of R-Au NCs, leading to sensitive ratiometric change. The effective and rapid visualization of the hPEI-SDS interaction is achieved based on DE-Au NC labeling, which endows the monitoring of the full process. On the basis of this proposed sensing nanoprobe, critical points, including CAC, CNC, and PSP concentrations of SDS, are easily observed using a fluorescence spectrophotometer. Our study not only proposes a ratiometric nanoprobe to visualize the hPEI-SDS interaction but also demonstrates a general approach to study polymer-small molecule interaction, and thus new avenues for the design of optical nanoprobes toward molecular interaction monitoring via the introduction of the in situ labeling strategy might open up in the analytical and material-related fields.

## Data Availability

Not applicable.

## Supplementary Materials

Supplementary Figures S1–S8 associated with this article can be downloaded at: https://www.mdpi.com/article/10.3390/bios12090686/s1, Figure S1: (A) Fluorescnece emission spectra of DE-Au NCs prepared with various hPEI amounts. (B) Fluorescence intensity of I_595_ (red line) and I_430_ (blue line) of DE-Au NCs versus the hPEI amount. (C) The intensity ratio (I_595_/I_430_) of DE-Au NCs versus the hPEI amount; Figure S2: (A) The temerature-dependent fluorescence intensity (I_595_) and intensity ratio (I_595_/I_430_) of DE-Au NCs produced by 0.25 μmol (A,D), 1 μmol (B and E), 11 μmol (C,F) hPEI, respectively; Figure S3: Time-resolved fluorescence decay spectra of DE-Au NCs under 20 °C (A), 45 °C (B), 70 °C (C), and return to room temperature (D), respectively; Figure S4: Hydrodynamic diameters of DE-Au NCs at 20 °C (A), 70 °C (B) and restored to room temperature (C), respectively; Figure S5: Temperature-dependent fluorescence emission spectra of FlS in the absence (A) and presence (B) of hPEI. Inset images are the plots of corresponding relative intensity (490 nm) versus the temperature; Figure S6: Hydrodynamic diameters of hPEI solution after addition of SDS with different concentrations at pH = 11 (black line), pH = 7 (red line) and pH = 3 (blue line), respectively; Figure S7: Time-resolved fluorescence decay spectra of DE-Au NCs solution with 0 mM SDS (A),1.6 mM SDS (B), 5 mM SDS (C) and 8 mM SDS (D); Figure S8: TEM images of DE-Au NCs solution with 1.6 mM SDS (A), 5 mM SDS (B) and 8 mM SDS (C).
