# Supplementary material for: Visualization of Polymer–Surfactant Interaction by Dual-Emissive Gold Nanocluster Labeling
Source: Biosensors (Basel). 2022 Aug 26;12(9):686. doi: 10.3390/bios12090686 (PMC9496207; doi:10.3390/bios12090686)
Supplement: Supplementary file 1 [file biosensors-12-00686-s001.zip › biosensors-1859188-supplementary.pdf]

Supplementary Material:

Visualization of Polymer-Surfactant Interaction by Dual Emissive Gold Nanoclusters Labeling

Jiaojiao Zheng[1], Jing Zhang[1], Fengniu Lu[2], Yi Du[3], Ding Cao[1], Shui Hu[1], Yang Yang[3,*] and Zhiqin Yuan[1,*]

[1] State Key Laboratory of Chemical Resource Engineering, College of Chemistry, College of Material Science and Engineering, Beijing University of Chemical Technology, Beijing 100029, China

[2] Department of Chemistry and Chemical Engineering Beijing Institute of Technology, Beijing, 100081, China

[3] Analysis Center, Key Laboratory of Bioorganic Phosphorus Chemistry and Chemical Biology (Ministry of Education), Department of Chemistry, Tsinghua University, Beijing 100084, China

[4] State Key Laboratory of NBC Protection for Civilian, Beijing 102205, China

* Correspondence: ricdyihui@163.com (Y. Yang)
yuanzq@mail.buct.edu.cn (Z. Yuan)

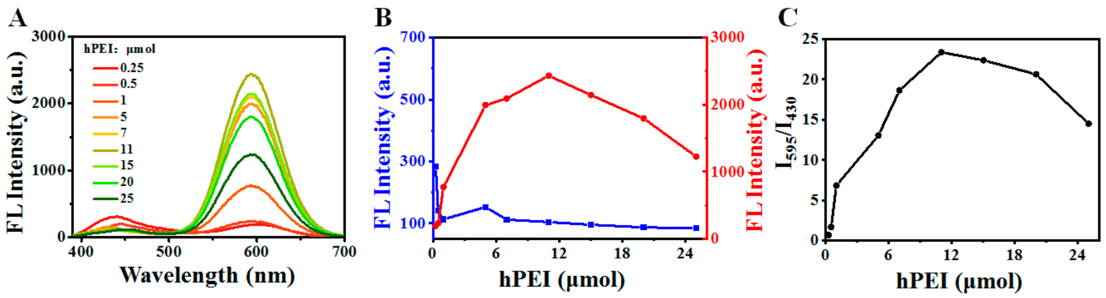

Figure S1. (A) Fluorescnece emission spectra of DE-Au NCs prepared with various hPEI amounts. (B) Fluorescence intensity of I_{595} (red line) and I_{430} (blue line) of DE-Au NCs *versus* the hPEI amount. (C) The intensity ratio (I_{595}/ I_{430}) of DE-Au NCs *versus* the hPEI amount.

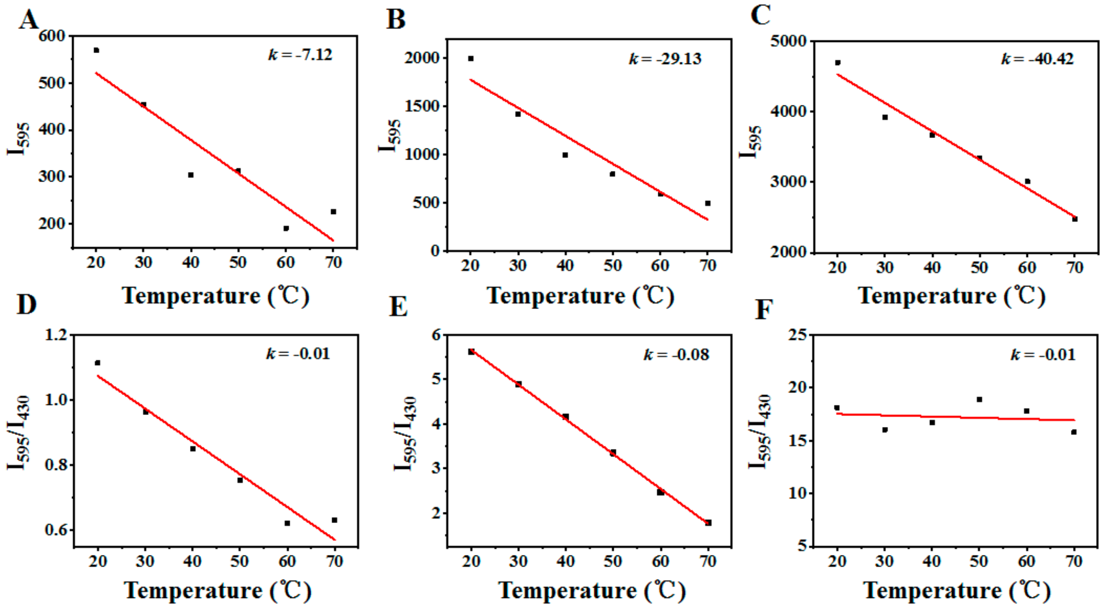

Figure S2. (A) The temerature-dependent fluorescence intensity (I595) and intensity ratio (I595/ I430) of DE-Au NCs produced by 0.25 μmol (A and D), 1 μmol (B and E), 11 μmol (C and F) hPEI, respectively.

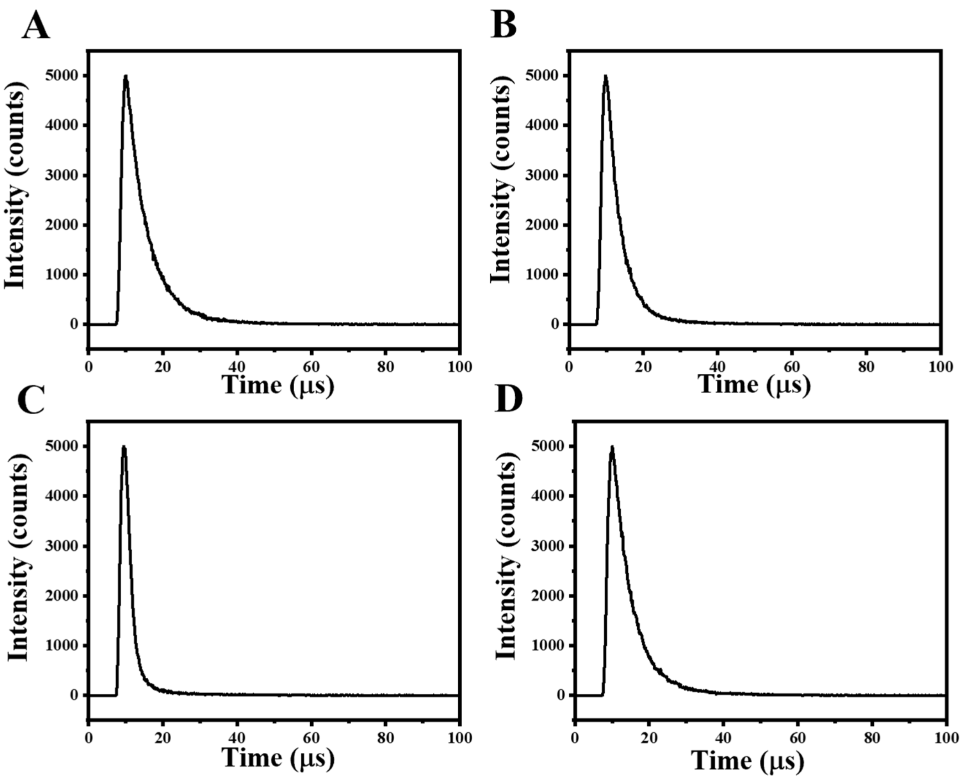

Figure S3. Time-resolved fluorescence decay spectra of DE-Au NCs under 20 ºC (A), 45 ºC (B), 70 ºC (C), and return to room temperature (D), respectively.

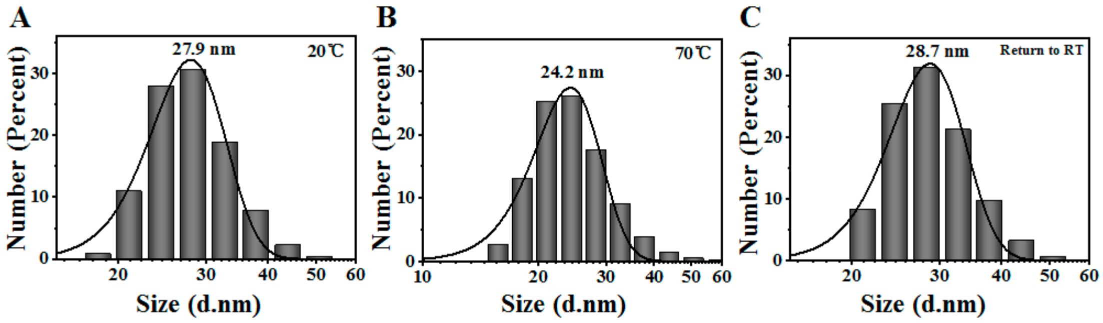

Figure S4. Hydrodynamic diameters of DE-Au NCs at 20 °C (A), 70 °C (B) and restored to room temperature (C), respectively.

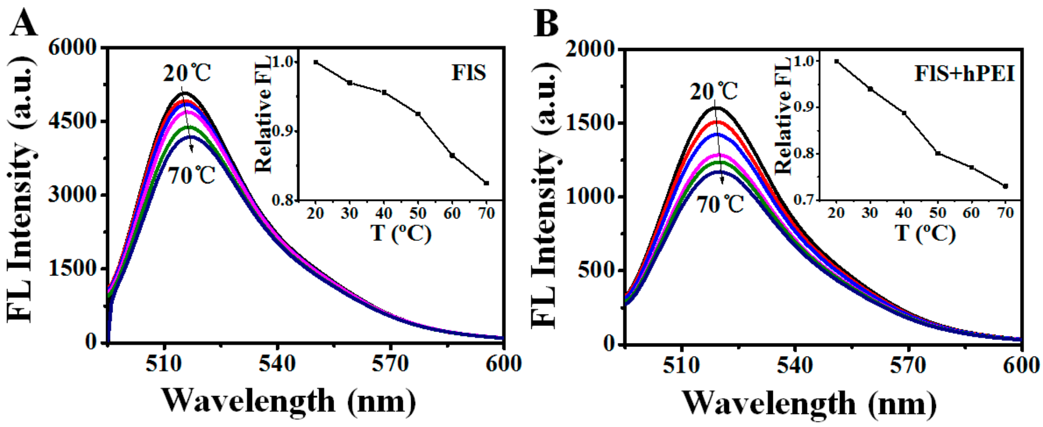

Figure S5. Temperature-dependent fluorescence emission spectra of FlS in the absence (A) and presence (B) of hPEI. Inset images are the plots of corresponding relative intensity (490 nm) *versus* the temperature.

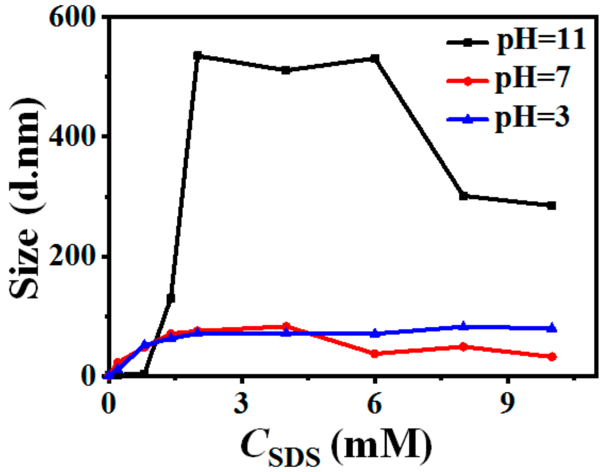

Figure S6. Hydrodynamic diameters of hPEI solution after addition of SDS with different concentrations at pH=11 (black line), pH=7 (red line) and pH=3 (blue line), respectively.

.

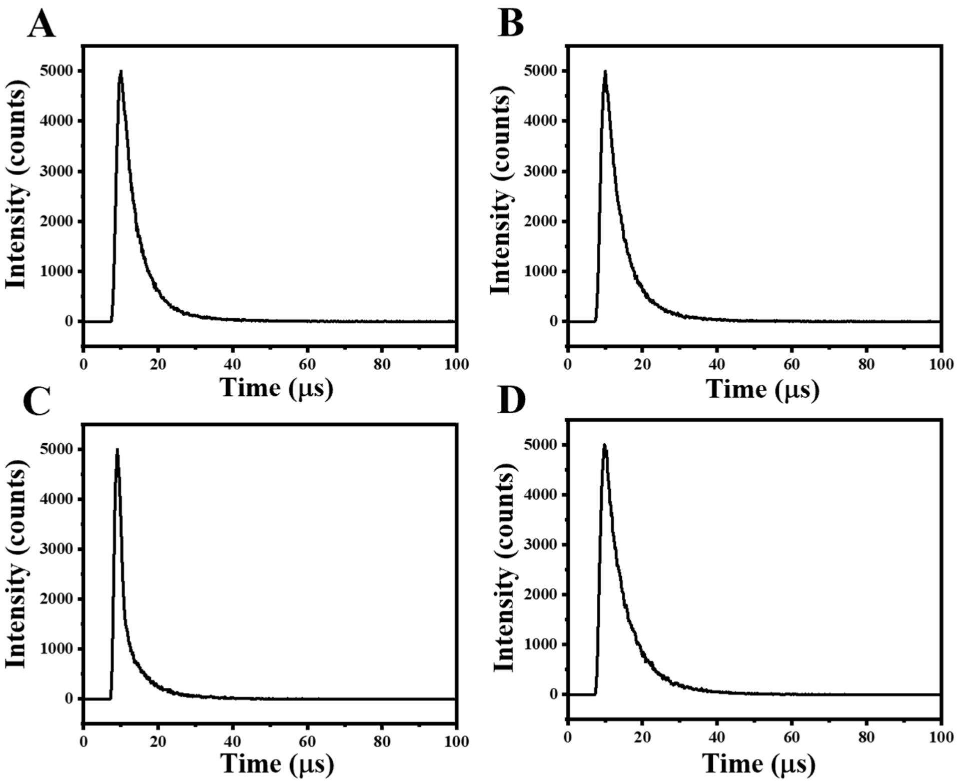

Figure S7. Time-resolved fluorescence decay spectra of DE-Au NCs solution with 0 mM SDS (A),1.6 mM SDS (B), 5 mM SDS (C) and 8 mM SDS (D).

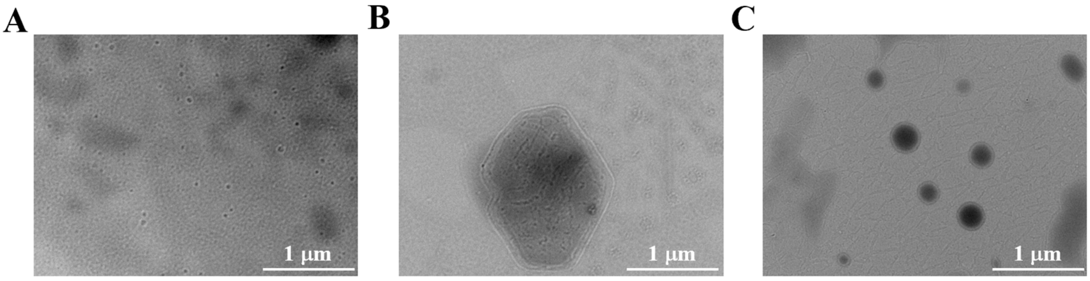

Figure S8. TEM images of DE-Au NCs solution with 1.6 mM SDS (A), 5 mM SDS (B) and 8 mM SDS (C).
